# The Punished Self, the Unknown Self, and the Harmed Self – Toward a More Nuanced Understanding of Self-Harm Among Adolescent Girls

**DOI:** 10.3389/fpsyg.2021.543303

**Published:** 2021-04-06

**Authors:** Line Indrevoll Stänicke

**Affiliations:** ^1^Department of Psychology, University of Oslo, Oslo, Norway; ^2^Nic Waals Institute, Lovisenberg Hospital, Oslo, Norway

**Keywords:** adolescence, lived experience, self-harm, self-identity, qualitative study

## Abstract

Self-harm among adolescents, mostly girls, has increased in the last years. Self-harm is associated with mental illness and the risk of suicide. This qualitative study aims to explore the lived experience of self-harm as it is related to everyday life and challenges among adolescents. Nineteen girls (13–18 years of age) in a clinical population (strategic selection) participated in personal interviews analyzed by Interpretative Phenomenological Analysis to capture how they made meaning of self-harm and essential features of experiencing self-harm. Adult persons with the first-hand experience of self-harm were included in the research analysis. Data-analysis resulted in three superordinate themes which all speak about ways to handle inner pain and vulnerability: 1. “I deserve pain,” 2. “I don't want to feel anything,” and 3. “I'm harmed, and no one cares.” Each superordinate theme included four main themes characterizing essential features of difficult experiences during self-harm, the purpose of the action, self-descriptions, and the role of others during self-harm. The three superordinate themes are discussed as emerging self-representations – “the punished self,” “the unknown self,” and “the harmed self” – during the transitional age of adolescence. This article argues that subjective personal data on self-harm related to adolescents' everyday lives may indicate diversity in the capacity to integrate difficult needs, feelings, and traumatic experiences as part of the self. This knowledge may bring a nuanced understanding of self-harm in adolescence, enhance self-understanding and treatment motivation, and inform clinical adjustment.

## Introduction

Self-harm usually starts in adolescence, with an onset of 12–13 years of age (Nock, 2014). The definition and concepts regarding direct self-harm are debated, particularly about whether to include or exclude suicidal intent. “Deliberate self-harm” refers to “intentional self-poisoning or self-injury, irrespective of the type of motive or extent of suicidal intent” (Hawton et al., 2012, p. 2373), and “non-suicidal self-injury” refers to “the deliberate destruction of one's bodily tissue in the absence of suicidal intent and for reasons not socially sanctioned” (Bentley et al., 2014, p. 638). Despite variations in terminology and definitions, self-harm has an estimated prevalence of 13–17% in community samples and up to 50–60% in clinical samples in several countries (Swannell et al., 2014; Gillies et al., 2018). The frequency has increased evenly since 1990 in several countries, and especially among young girls (Gillies et al., 2018; Morgan et al., 2018). Cutting is the most common method of direct self-harm among both genders, but girls report up to five times more direct self-harm compared to boys who report more indirect forms (e.g., involvement in risk situations) (Nock, 2014). How can we understand the high frequency of self-harm among adolescents and especially among young girls?

On a group level, findings from epidemiological studies show that self-harm is associated with a range of mental illnesses, an increased risk of suicide, and several risk factors including sociodemographic characteristics, negative life events and trauma, and genetic, psychiatric, and psychological factors (Miller et al., 2019). Although several theoretical models have been suggested to explain the function of self-harm (Soyemoto, 1998), including developmental and interpersonal perspectives, a review of empirical research on self-harm found the strongest support for self-harm as “an affect regulation function” (Klonsky, 2007; Miller et al., 2019: Nock, 2014). Self-harm reduces overwhelming and difficult feelings and brings relief and control. However, most reviews focus on adult samples or non-clinical adolescent samples, and primarily including data on the function of self-harm from questionnaires. The states, intentions, individual variables, and answer categories are in these studies predetermined by the researchers. The complexity of the phenomenon of self-harm may require studies with different research methods, different age-groups, and both non-clinical and clinical samples, to add theoretical concepts and increase knowledge for further research. Qualitative studies, highlighting knowledge development and theory building from personal descriptions of a phenomenon, may contribute to a nuanced understanding of self-harm in adolescence.

There is a need for more knowledge to understand the high frequency among young girls, and also, as earlier mentioned, that self-harm is associated with a range of different risk factors and mental illnesses. There is also a great diversity among self-harming adolescents – some quit after a few attempts, some after a few years, and others continue self-harming into adulthood with extensive mental difficulties. Also, to regulate affects, could self-harming practices be ways of using one's body to handle the developmental challenges of self-representation and interpersonal problems without bothering others – as an attempt to reach autonomy?

This qualitative study aims to explore the subjective lived experience of self-harm among adolescents in a clinical population through personal interviews and to enhance knowledge of the essential features of the meaning-making of their behavior. The research questions were: Firstly, how do adolescents in a clinical sample describe experiences of their self-harm? Secondly, are there essential features of how adolescents make meaning of their self-harm? This study can enhance knowledge on how self-harm is related in different ways to mental illness, and how experiences of self-harm may indicate diversity in the capacity to integrate difficult needs, feelings, and traumatic experiences as part of the self.

### Understanding Self-Harm

In several clinical case studies, authors have emphasized how self-harm may be understood as a way to handle and cope with trauma (Gardner, 2001; Turp, 2003; Yakeley and Burbridge-James, 2018). Further, these authors underline how self-harm may have several functions – to regulate affects, to handle memories related to trauma, and to communicate mental content that cannot be expressed with words. Self-harm is described metaphorically as “the voice of the skin” (McLane, 1996). Further, the interpersonal function is highlighted by labeling self-harm as “a sign of hope” for change (Motz, 2010), or an effort to “cut the silence” and break out of an insufficient emotional and familial circumstance (Brady, 2014). However, case studies are often based on adult patients, seldom include the patients' quotes, and are often theory-driven (Klonsky, 2007; Nock, 2014; Stänicke et al., 2018).

Some qualitative studies explore systematically the experience of self-harm with open or less structured interviews. In a systematic qualitative study of the experience of self-harm among young adults, self-harm is underlined as misunderstood by most people and a way to stay alive (Brown and Kimball, 2012). Sinclair and Green (2015) also underline how adult patients related self-harming to adolescent distress, misuse of alcohol, and that self-harm was a symptom of untreated or unrecognized illness during adolescence. Many qualitative studies focus on adult patients with a borderline personality disorder or non-clinical samples of young adults (Stänicke et al., 2018). Further explorations of how adolescents integrate and give meaning to their self-harm might bring us closer to an understanding of how this behavior becomes an important part of many young peoples' life during the transition to adulthood.

Some qualitative studies highlight how adolescents experience self-harm in an interpersonal context. Adolescents relate self-harm to psychological pain and anger in close relationships (Abrams and Gordon, 2003), and they associate their beginning of self-harm with interpersonal stressors (McAndrew and Warne, 2014). Difficulties with family and friends may be the reason for self-harming and, still, family and friends are important to get help and support to end self-harming (Gelinas and Wright, 2013; Wadman et al., 2018). According to a recent meta-synthesis on 20 qualitative studies of self-harm from adolescents' perspective, young people experience self-harm as an attempt to (1) obtain release and to (2) control difficult feelings, but also to (3) represent unaccepted feelings, and to (4) connect with others (Stänicke et al., 2018). Self-harm is discussed as a function of affect regulation but also as an attempt to *express* or *share* important conflictual emotional and relational information from the adolescent's private life.

Several of the included qualitative studies in the mentioned meta-synthesis included non-clinical samples. In a study by Grandclerk et al. (2019), including informants from a clinical sample, relational dimensions appeared as common aspects of the subjective experience of both self-harm and suicide attempts among young women. These young women described self-harm and suicide attempts in close relationships with the self, with others, with their bodies, and with death. Following these findings, Grandclerk et al. (2019) discuss self-harm as related to complexities in the psychological developmental issues of separation-individuation during adolescence, and that self-harm may be an attempt to achieve autonomy for these youths. Hetrick et al. (2020) highlight the importance of the idiosyncratic nature of triggers and self-help strategies and “the need for young people to maintain some autonomy and control while being supported to connect with others for support” (p. 1). They found several subjective experienced precipitating factors and triggers for self-harm which may increase knowledge of their need to self-harm, such as distressing emotions, sense of isolation, exposure to self-harm, relationship difficulties, social comparison, and school/work difficulties. Another recent qualitative study, Stänicke et al. (2019) highlights how young girls in a clinical sample experience common aspects *and* different pathways into and out of self-harm. All girls began self-harming because of difficult feelings and relational problems but described their problems differently – as a way to handle self-criticism, diffuse stress, or earlier traumatic events. They were all ambivalent to treatment and to end self-harming but emphasized to explore self-harming situations with their therapist. Interestingly, regarding autonomy as a possible developmental challenge during adolescence, they underlined how they discovered their own way out of self-harm. Further, they described different ways out of self-harm – by being understood and developing self-supporting monologs, by sharing experiences and trying coping-strategies, or by being respected and receiving practical support (Stänicke et al., 2019).

Some studies of sub-groups among people who self-harm have focused on differences in the *frequency of self-harm* (Klonsky and Olino, 2008; Whitlock et al., 2008; Somer et al., 2015; Xin et al., 2016), *symptoms of mental illness* (Ross and Heath, 2003; Stanford et al., 2016), *psychosocial adjustment* (Hamza and Willoughby, 2013), or *methods of self-harm* (Andover et al., 2005; Bracken-Minor et al., 2012). For example, Andrewes et al. (2017) included structural interviews and found a higher risk of suicide attempts among adult patients with borderline personality disorder and among those with random and impulsive self-harm rather than habitual. Still, most studies on sub-groups include personal data from questionnaires on adults or young adults and mostly involve non-clinical samples. An analysis of the essential features of adolescents' experience of self-harm may add information on important diversities among self-harming adolescents.

Adolescence is a life transition period is characterized by cognitive, biological, psychological, and social changes (Siegel, 2015; Thapar et al., 2015). During adolescence, young girls and boys may develop an increased capacity for problem-solving, affect regulation, and relational reciprocity toward a mature identity and representation of the self (Erikson, 1980; Luyten et al., 2020). Emotional arousal, impulsivity, difficult feelings, and interpersonal stress are often overwhelming, and risk behaviors are not uncommon. As mentioned, the function of self-harm has been discussed according to developmental challenges during adolescence in theoretical models (Soyemoto, 1998; Lemma, 2010). According to Straker (2006), the act of harming the body may be a sign of a developmental disturbance or as a deficit in the self-structure. Self-harm may be an attempt to put in place elements involved in the building of a sense of self, like mirroring, establishing boundaries between self and others, and a narrative autobiographical memory. Le Breton (2017) also describes how the body becomes the battlefield of self-identity among adolescents, and how the body may be experienced as an object, different from self, which can be dealt with, punished, or disciplined through physical attacks. Rossouw and Fonagy (2012) discuss how self-harm expresses disturbances in the capacity of “mentalization” – a capacity to understand one's own and other's behavior as mental states. In their perspective, the development of mentalization is essential for affect integration, for impulse-control, to distinguish self from others, and to have a coherent self-representation. Especially during adolescence, cognitive, emotional, and relational developmental challenges may influence the young person's capacity to represent and understand their own and others' behavior (Luyten et al., 2020).

Results from case studies and qualitative studies, and different theoretical concepts, point to the value of studying adolescents' experience of self-harm from their perspective, to explore essential features of their meaning-making, and to explore how self-harm *in different ways* may be related to developmental and interpersonal issues. This knowledge is valuable to clinicians to detect and support vulnerable youths, to help them feel understood, and to make a hypothesis of adjustments in clinical interventions.

## Materials and Methods

In this qualitative study, the main focus was to explore the participants' subjective experience of self-harm as part of their daily life. The sample size and data analysis were chosen to get rich and nuanced descriptions of the adolescents' life world. The informant and the researchers' perception and empirical data were acknowledged as selective, partial, and context-dependent, and the findings should therefore be related to the social and cultural context (Stiles, 1993; Levitt et al., 2017). The most important methodological challenge was to reach the trustworthiness of the findings and to develop theoretical concepts and knowledge more than objectivity and generalizable data. However, the developed concepts can be discussed according to results from other studies on self-harm with other samples and in other contexts.

### Study Setting and Participants

#### Sample and Interviewees

The sample consisted of 19 girls whose initial treatment contact at an outpatient clinic in Norway, offering treatment for children and adolescents (0–18 years of age) free of charge, suggested self-harm (impulsive or repetitive, with or without suicide intentions). Interviewees were selected as *informative exemplars* for a *purposive sample* rather than a representative sample, to be good examples to explore subjective experience and to nuance differences in the phenomenon of self-harm. The selection of cases depended partly on the inclusion of earlier cases to secure *sample-heterogeneity* related to age, gender, frequency and form of self-harm, symptom disorder, personality disorder, socio-economic, and cultural background, and education. Their age ranged from 13 to 18 years (mean = 15.9 years). Nine girls lived with both parents or part-time with their mother and father. Six girls lived with their mothers and had seldom or no contact with their fathers. Four girls were in foster care and joint school only part-time. Six girls had an earlier treatment history. They were recruited for personal interviews in their first course of treatment (up to 6 months into treatment). On average, the girls began self-harming at 13.1 years of age and harmed themselves 1–3 times per week during the prior year. They harmed themselves mainly by cutting, mostly in combination with other methods like scratching, hitting, and burning. All participants confirmed thoughts of suicide and nine reported one or several suicide attempts. The participants met diagnostic criteria for a range of disorders, mostly mood disorders, anxiety disorders, and eating disorders, and 13 met criteria for a personality disorder, most often avoidant, borderline, and depressive type.

#### Researchers

The main researcher and interviewer, LIS, is a clinical psychologist and a researcher at the University of Oslo, Norway. LIS collaborated with two co-researchers, HH and SEG, throughout the research process, and met regularly to discuss and reflect upon the research process, data analysis, and results. Both HH and SEG are researchers and clinical psychologists. The researchers represented different methodological and theoretical backgrounds, with respectively relational dynamic therapy and integrative psychotherapy. All are engaged in psychotherapy research.

#### Service User Involvement

Three persons with the first-hand experience of self-harm were included in a reference group. They were involved through all stages of the research process by discussing research questions, interview guides, and emerging themes. Their comments on interview questions and the presentation of results in the manuscripts were of importance to increase the ecological validity of the study.

### Procedures

#### Recruitment

Therapists at the clinic were informed about the project and asked potential participants. In this study, a purposive sample was chosen (see Sample and Interviews). Although both girls and boys were invited, some fewer boys confirmed self-harm to their clinicians, and they described more indirect self-harm. The prevalence is also highest among girls. Data were collected from 19 girls (13–18 years of age) who consented to participate and were interviewed over 1.5 years.

#### Personal Interviews

The adolescents participated in personal interviews that followed a lightly structured and open-ended interview-guide, developed by LIS, co-researchers, and the reference group. The interviews were open and lightly structured to explore the participants' subjective experience of their lifeworld, and to capture personal nuances and rich descriptions of concrete experiences of self-harm as part of their practices with family and friends, at home or school or other important places for them. The interviewer, LIS, asked about when they started to self-harm and invited them to describe events from the day before the interview and how practices of self-harm took place in their everyday life [inspired by the *Life-mode interview* (Haavind, 2019)]. The interviewer explored feelings, thoughts, and actions in difficult situations related to self-harm the day before and in the last self-harming episodes to detect how they experienced and gave meaning to their self-harm. The adolescents participated in two personal interviews, 60–90 min each, within a 2–4 weeks period. The interviewer articulated her understanding during the interviews (empathically and through questions) which allowed the informant to explore self-harming with a reflexive listener and an opportunity to confirm, nuance, or correct. Interviews were anonymized, audio-recorded, and transcribed. They received a gift card (20 USD) for participating in the study.

### Data Analysis

The qualitative data-analysis was grounded in a phenomenological hermeneutical epistemology and specifically informed by *Interpretative Phenomenological Analysis* (IPA; Smith et al., 2009) aiming to both explore the adolescents' experiences of self-harm and to interpret meaning from the participants' descriptions to develop concepts. IPA emphasize a phenomenological perspective of how events are perceived and experienced by individuals in a social and cultural world, and a hermeneutic perspective in underlining how observations and experiences always are interpretative. IPA was chosen because this approach is particularly appropriate both to describe the semantic content of the participants' experiences *and* to understand the complexities of the participants' experience and interpret the meaning of self-harm concerning psychological processes (Smith et al., 2009). It was significant to choose a qualitative method to explore and analyze perspectives, perceptions, and meaning-making of adolescent's lifeworld through personal interviews, while also relating these descriptions to already established knowledge and theories on self-harm. Importantly, IPA explicitly describes procedures for a phenomenological study on ideographic material while interpreting the data material hermeneutically according to the knowledge of mental processes. In this way, the data was descriptive *and* interpreted by the researcher and the co-researchers. To enhance the quality of the data analysis, we followed the IPA quality evaluation guide (Smith, 2015).

The data-analysis consisted of several phases, primarily conducted by the author of this article but in every phase presented and discussed with co-researchers (HH and SEG) to minimize bias (investigator triangulation; Flick, 2002). Firstly, we read and reread the transcribed text to become familiar with the data. We became aware of how differently the participants described and integrated experiences of self and others during self-harm and decided to analyze this topic in detail. Secondly, parts of the texts related to the experience of self and others during self-harm were separated into broad content meaning for each participant. Thirdly, emerging sub-themes and themes for each case were suggested before content was compared and developed across the sample. Selected quotes were kept enhancing transparency. At this level, we moved from the descriptive level to a higher level of abstraction and theoretical framework and looked for convergences, divergences, and patterns in the content as well as essential features of how the participants described their self-harming. The analysis was in this way based on the participants' terminology in the transcripts but the interpretation of the data was involved in an ongoing process which may be described as “the hermeneutic circle” – looking at the part and the whole and back again (Smith, 2015). Although all interviews supplied the analysis with unique data, the interpretations of the first 10 cases encompassed more or less the next nine cases. In the end, the emergent themes and associated sub-themes concerning essential aspects of the experience of self and others during self-harm across the participants were organized into three superordinate themes capturing three ways of experiencing self-harm and to handle inner pain and vulnerability.

The research group worked with the guiding principle of capturing the complexity of the data, which helped the main researcher to become self-reflective and aware of different ways of reading the data (Levitt et al., 2017). Still, the author of this article presented preliminary codes, repeating ideas, and preliminary interpretations of the participants' experiences of self-harm, and the organization of the data and the descriptive labels were discussed, which ended in agreement (all three agreed) or became integrated into nuances of the material (one or two disagreed), such as renaming, rearranging, adding or merging themes or sub-themes. During the data analysis, there were systematic procedures for linking interpretations with concrete observations or data to ensure that the results were rooted and grounded in rich exemplars of data and which allows the reader to judge the fidelity of the analysis (Levitt et al., 2017).

### Credibility Checks and Reflexivity

Throughout the research process, several credibility checks were included to ensure the trustworthiness and methodological integrity of the data and results (Levitt et al., 2017). During the interviews and in feedback meetings, the informants were allowed to add and nuanced information. During the analysis process, the researchers emphasized being aware of personal and theoretical perspectives, made individual discussion notes, and engaged in self-reflection to increase reflexivity and reduce researcher bias. Although LIS primarily organized the content into themes and sub-themes, the diverse interpretations of data were noted in research meetings with SEG and HH, discussed into an agreement, or integrated into meaningful nuances of the material, such as renaming, adding, or merging themes or sub-themes (Levitt et al., 2017). Because of the active role of the researchers in the organization and interpretation of data, reflexivity to personal responses and biases were important to remain aware of during the movement from the participants' descriptions of experience and into our interpretations of their experiences. The three persons with a user perspective read the interview guide and manuscript, expressed how they recognized nuances in the findings, and had helpful comments on the language. LIS wrote the manuscript. SEG and HH read the manuscript.

#### Ethics Approval and Consent to Participate

The adolescents and their parents received written and oral information from their therapist and the interviewer and gave written consent to participate in the research study and for publication following the Declaration of Helsinki. To secure comfort and support for the participants while talking about sensitive topics in the interview, the interviewer was conducted by LIS who is a trained clinician and had worked at the clinic for 10 years and could provide support if the risk was detected. The participants received treatment at the clinic, which made sufficient interventions available. LIS had no role in the participants' treatment but informed the therapists about high suicide risk on three occasions. The Norwegian Regional Committees for Medical and Health Research Ethics approved the study (2014/832). All interviews were anonymized before data-analysis. Participants approved selected and anonymized quotes.

## Results

**Figure 1:**
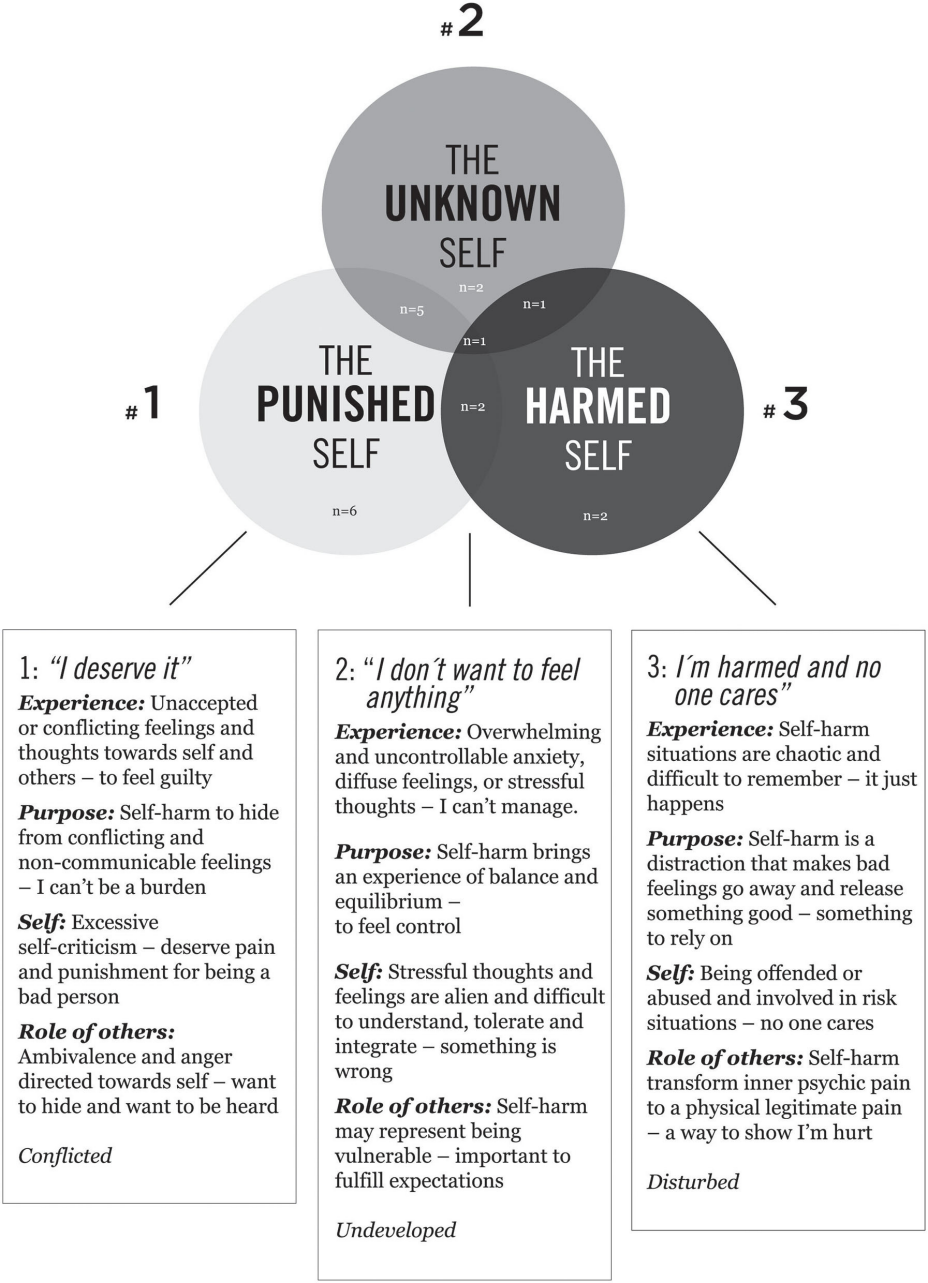
Three superordinate themes and associated themes convey adolescents' lived experience of self-harming.

The data analysis resulted in three superordinate themes encompassing four main themes and several sub-themes, which represent experiences of self-harm, and interpreted the meaning of the participants' experiences (see Table 1). More specifically, the associated themes are highlighting essential features of (1) difficult experiences during self-harm, (2) experienced the purpose of the action of self-harm, (3) self-descriptions, and (4) the role of others. In the following, three selected participants – Mary, Lisa, and Sophie – illustrate each of the three superordinate themes with associated themes and sub-themes and exemplified by quotes. The selected participants represent especially rich data, and illustrate qualitative divergences, both in description and integration of their experiences and in interpretations of their experiences. The superordinate themes, themes, and sub-themes are rooted in the data-analysis of all participants, and each superordinate theme and associated themes represent different participants (respectively 6, 7, and 6 informants) (see Figure 1). However, some of the associated sub-themes represent some participants more than they represent others (presented and enumerated in Table 1). Eight participants covered a few sub-themes from one of the other superordinate themes, and one person covered sub-themes from all (see Figure 1).

**Table 1:** Three superordinate themes with associated themes and sub-themes.

**Superordinate themes**	***Themes***	***Sub-themes (n = #1/#2/#3)***
**#1: The punished self – I deserve it (*****n*** **=** **6)** Self-harm may express self-punishment and represent both self-criticism and frustration of being restricted in life.	***1.1 Experiences during self-harm:*** Unacceptable or conflicting feelings and thoughts toward self and others – I feel guilty	*1.1.1 Sadness and envy (n = 6/4/0)* *1.1.2 Feeling dull and boring (n = 4/0/0)* *1.1.3 Irritated and angry (n = 6/1/0)* *1.1.4 The feelings make them feel guilty about a close person (n = 6/0/0)*
	***1.2 Purpose of action:*** Self-harm to hide from conflicting and non-communicable feelings – I can't be a burden	*1.2.1 Unfamiliar with expressing feelings – especially anger (n = 4/0/0)* *1.2.2 Attend to how people think of them (n = 4/1/0)* *1.2.3 Important to not be a burden (n = 3/0/0)* *1.2.4 A tight daily schedule helps them to keep busy and to feel valuable (n = 3/0/0)* *1.2.5 Sad and frustrated or angry because they haven't achieved their goals (n = 3/1/0)*
	***1.3 Self-description:*** Excessive self-criticism – deserve pain and punishment for being a bad person	*1.3.1 Excessive self-criticism and a feeling of not being worthy (n = 5/1/0)* *1.3.2 Everyone thinks they are horrible (n = 4/1/3)* *1.3.3 Self-blame – deserve pain and punishment for being a bad person (n = 4/1/1)* *1.3.4 Self-harm as relief, satisfaction and a way to attend to something else (n = 4/0/0)* *1.3.5 Thoughts of suicide during self-harm* – *to get rid of all the demands (n = 3/1/2)*
	***1.4 Role of others:*** Ambivalence and anger directed toward self – want to hide and want to be heard	*1.4.1 Self-harm as a way to express their feelings or having problems (n = 3/0/0)* *1.4.2 Do not express their feelings but want to be heard and attended to (n = 3/0/0)* *1.4.3 Can't live without self-harm even if it causes problems and conflicts (n = 3/0/0)* *1.4.4 Self-harm as a request for others to listen (n = 1/0/0)*
**#2: The unknown self – I don't want to feel anything(*****n*** **=** **7)** Self-harm may represent how unknown, unacknowledged, or incomprehensible experiences are cut off and, at the same time, may evoke curiosity for being vulnerable.	***2.1 Experiences during self-harm:*** Overwhelming and uncontrollable anxiety, diffuse feelings, or stressful thoughts – I can't manage	*2.1.1 Difficult to describe what happens before, during or after self-harm (n = 0/ 3/ 0)* *2.1.2 Overwhelming stress and anxiety (n = 0/ 5/ 0)* *2.1.3 Feelings are experienced as a problem – too much or too little (n = 0/5/0)* *2.1.4 Overwhelming negative thoughts (n = 0/6/1)*
	***2.2 Purpose of action:*** Self-harm brings an experience of balance and equilibrium – to feel control	*2.2.1 Incomprehensible feelings become concrete – something to focus on (n = 0/4/0)* *2.2.2 Self-harm brings relaxation (n = 0/5/0)* *2.2.3 Self-harm gives a sense of control and to manage something (n = 0/4/0)* *2.2.4 Suicide thoughts are not related to self-harm – do not want to die (n = 0/3/0)*
	***2.3. Self-description:*** Stressful thoughts and feelings are alien and difficult to understand, tolerate, and integrate – something is wrong with me	*2.3.1 Hate the feeling of not understanding – feeling insecure and stupid (n = 0/4/0)* *2.3.2 Difficult feelings are experienced as alien – out of the blue(n = 0/4/0)* *2.3.3 A painful state of helplessness and being out of control (n = 0/4/0)* *2.3.4 Don't know any other ways to find calmness (n = 0/3/0)*
	***2.4 Role of others***: Self-harm may express being vulnerable – important to fulfill expectations	*2.4.1 Hard to fulfill expectations – have to be best, proper and clever (n = 0/3/0)* *2.4.2 Problems and self-harm must be kept hidden – no one should know (n = 0/3/2)* *2.4.3 If given the opportunity, they may detect feelings related to self-harm (n = 0/3/0)* *2.4.4 Miss to speak more openly (n = 0/2/0)* *2.4.5 Self-harm as a sign of being vulnerable and in need of help (n = 0/4/0)*
**#3: The harmed self – I'm harmed, and no one cares(*****n*** **=** **6)** Self-harm may be a concrete and unrepresented expression of being invaded and assaulted.	***3.1 Experiences during self-harm:*** Self-harm situations are chaotic and difficult to remember – it just happens	*3.1.1 Hard to remember situations leading to self-harm (n = 0/0/3)* *3.1.2 Chaotic feelings and thoughts about self and others (n = 0/0/6)* *3.1.3 A feeling of being left alone (n = 0/0/6)* *3.1.4 Self-harm is not related to anything specific in their life (n = 0/0/2)*
	***3.2 Purpose of action:*** Self-harm is a distraction that makes difficult feelings go away and release something good – something to rely on	*3.2.1 A distraction by attending to the blood and the pain (n = 0/0/3)* *3.2.2 Makes bad feelings, anxiety, and thoughts go away (n = 5)* *3.2.3 Releases something good (n = 0/2/3)* *3.2.4 Could make you addicted (n = 0/2/4)* *3.2.5 Self-harm brings back a feeling of safety (n = 0/0/3)*
	***3.3 Self-description:*** Being offended or abused and involved in risk situations – no one cares	*3.3.1 They describe experiences of violence, assault and abuse (n = 0/0/4)* *3.3.2 Being involved in risk situations as a child (n = 0/0/3)* *3.3.3 Being involved in risk situations today and don't know why (n = 0/0/3)* *3.3.4 Carelessness and lack of self-support in difficult situations (n = 0/0/4)* *3.3.5 Suicidal thoughts always in the background (n = 0/0/3)*
	***3.4 Role of others***: Self-harm transform inner psychic pain to physical legitimate pain – a way to show I'm hurt	*3.4.1 Self-harm changes psychic pain (n = 0/0/4)* *3.4.2 A way to tell someone about their difficulties (n = 0/0/2)* *3.4.3 Parents saw their struggle or self-harm but didn't act (n = 0/0/2)* *3.4.4 Reactions to self-harming evoke reflections – it should not be this way (n = 0/0/3)*

### Superordinate Theme #1: The Punished Self – I Deserve It

The first superordinate theme and associated sub-themes represent an experience of self-harm dominated by self-criticism because of unacceptable and conflicting yet differentiated feelings, thoughts, and needs. Concretely, self-harm may express how pain is directed toward the body as punishment instead of being angry, assertive, or defensive toward others. Self-harm is experienced as deserved, and besides, may represent a frustration of being restricted in life. This experience during self-harm is labeled “the punished self.”

#### Experiences During Self-Harm: Unacceptable or Conflicting Feelings and Thoughts Toward Self and Others – I Feel Guilty

Mary (15 years of age) struggled with an eating disorder and harmed herself, primarily by cutting or banging her head, since she was 13 years old. During the last year, she harmed herself once a week. She described difficult feelings like sadness and envy (1.1.1) and feeling dull and boring (1.1.2) concerning her mother and brother: “It's something with my brother and mom (…) I wonder if mom just wanted one child … Maybe she would like me better if I was ill – maybe she thinks I am boring?” Further, Mary described being irritated and angry (1.1.3) at her mother, but her feelings made her feel shame and guilty (1.1.4): “I became a little irritated at my mom, but also ashamed because I can't get irritated at mom because she has her OWN opinions, and I can't be angry at mom just because she doesn't care about me. That is selfish and very egoistic, and I feel a lot of shame.” The essence of this theme is summarized as unacceptable or conflicting feelings and thoughts toward self and others – I feel guilty (1.1).

#### Purpose of Action: To Escape or Hide From Conflicting or Non-communicable Feelings – I Can't Be a Burden

Mary was unfamiliar with expressing her feelings and especially being angry (1.2.1): “Self-harming is the only way I show my feelings. Someone told me it could have been good for me to be angrier or scream more... but it's just not me.” It was important for Mary to attend to how people think of her (1.2.2) not be a burden (1.2.3): “I'm very attended to what people think of me, how they look at me if they get tired of me if I'm a burden or what kind of thoughts they have of me.” Mary made herself a very tight day-schedule (1.2.4) with schoolwork, exercises, food-plan, reading, being with her mother, and relaxing, which kept her busy and helped her to accomplish her goals and feel worthy. She got angry if she could not achieve her goals and harmed herself (1.2.5): “I don't want to bother people.” The action of self-harm is interpreted as a way to escape or hide from conflicting or non-communicable feelings – she can't be a burden (1.2).

#### Self-Description: Excessive Self-Criticism – Deserve Pain and Punishment for Being a Bad Person

Mary described excessive self-criticism and an intrusive feeling of not being worthy (1.3.1): “The feeling of not being worthy, it is so strong.” The feeling of being not good enough for her mother made her feel horrible or boring (1.3.2). Therefore, Mary harmed herself as a punishment for being who she was – a bad person (1.3.3): “When I have eaten too much … I become sad and feel a pressure to harm myself. I MUST do it because I have been bad, in a way.” Self-harm brought relief (1.3.4): “I think a lot about the cutting, it is a relief to think about something else … pain instead of psychic pain …. mm … more practical … cover it, clean up and then pretend it did not happen.” She confirmed thoughts of suicide-related to self-harm (1.3.5): “When I harm myself, I can pretend the cutting is to kill myself.” The essence of experiencing self during self-harm is summarized as excessive self-criticism - deserve pain and punishment for being a bad person (1.3).

#### Role of Others: Ambivalence and Anger Directed Toward Self – Want to Hide and Want to Be Heard

Mary described self-harm as a way to express difficult feelings or having problems (1.4.1): “It's like … to harm myself and not eat are ways I can express having a hard time.” Even if Mary did not want to express her feelings because she was afraid of being a burden, she also wanted to be heard (1.4.2). If she harmed herself and no one reacted, she became very disappointed: “It's always those who scream out loudest who get the most support. I think the silent persons also should be taken seriously.” She had a wish to find other ways to express her problems. Still, it was difficult. She described how she could not live without self-harm even if it causes problems and conflicts (1.4.3): “If I had to stop self-harming, it would have been like losing a part of me.” Self-harm was like a request for others to listen (1.4.4.) and brought Mary the security of being remembered: “If I can't harm myself – there are no reasons for people to show that they care about me, and then I get insecure if what I do is good enough.” Mary wished people could listen to what self-harm express: “People should be interested in what's BEHIND self-harm. Maybe I use self-harm... to get someone curious and worried.” The essence of the role of others during self-harm may be summarized as ambivalence toward expressing difficult thoughts and feelings toward others – want to hide and want to be heard (1.4) – and anger is interpreted as directed toward self instead of others.

### Superordinate Theme #2: The Unknown Self – I Don't Want to Feel Anything

The second superordinate theme and associated themes represent an experience of self-harm dominated by a struggle against feelings in general. Self-harm becomes a way to re-establish a sense of control, mastery, and balance over overwhelming stressful thoughts, anxiety, and diffuse feelings. Concretely, self-harm may represent how unknown, unacknowledged, or comprehensible experiences are cut off and, at the same time, may evoke curiosity for being vulnerable. This experience during self-harm is labeled “the unknown self.”

#### Experiences During Self-Harm: Overwhelming and Uncontrollable Anxiety, Diffuse Feelings, or Stressful Thoughts – I Can't Manage

Lisa (16 years of age) started to harm herself at 14 years old. She harmed herself by cutting 3–7 days a week, but sometimes by taking painkillers, pinching, or burning herself. She described feeling anxious and afraid since early childhood, but in the last 2 years, she experienced painful panic attacks. For Lisa, it was difficult to describe what happened before, during, or after self-harm (2.1.1): “In a way – it was – I cried a little and... it was very difficult, I don't know what it was... I was TOTALLY out.” She described overwhelming stress and anxiety (2.1.2): “I was stressed and – and really – desperate and in a way. I thought: Oh my God, how can I manage to finish this day.” In general, feelings were experienced as a problem – either too much or too little (2.1.3): “I can't manage feelings at all.” She also underlined overwhelming negative thoughts (2.1.4): “I can't stop worrying.” The essence of this theme is summarized as overwhelming and uncontrollable anxiety, diffuse feelings, or stressful thoughts – I can't manage (2.1).

#### Purpose of Action: Self-Harm Brings an Experience of Balance and Equilibrium – To Feel Control

Lisa explained how self-harm made incomprehensible inner pain and feelings concrete and gave her something to focus on (2.2.1): “Self-harm collects those feelings. Sometimes, when many painful things happen, and you can't understand … you just feel so much at once, it's like a storm – it's much easier to collect it all in ONE physical pain.” Self-harm brought a state of relaxation (2.2.2) and gave her a sense of control by managing something (2.2.3): “I was so stressed, and I didn't know what to do with myself. And then I did it to handle it (the stress)... to finish, in a way... Yes, 's like a control-thing … makes me relaxed.” She felt life, in general, was difficult and, occasionally, she would wish to become involved in an accident: “Sometimes I can imagine that the bus becomes involved in an accident … what if I get hurt.” In her experience, these thoughts were not related to self-harm (2.2.4): “I'm never going to kill myself... It would have made it easier, but I won't give up – I know someone loves me.” The action of self-harm is interpreted as a way to bring an experience of balance and equilibrium – to feel control (2.2).

#### Self-Description: Stressful Thoughts and Feelings Are Alien and Difficult to Understand, Tolerate, and Integrate – Something Is Wrong With Me

Lisa underlined how she disliked the feeling of not understanding because it made her feel insecure and stupid (2.3.1). These difficult feelings were experienced as alien – out of the blue (2.3.2) – like something she had to get rid of: “I just can't understand how I feel. I just feel so much at ONCE. And I'm just so in despair and frustrated. I don't know how to manage and at the same time I'm madly stressed and afraid of something, but I don't know what it is.” She experienced that something was wrong and described a painful state of being helpless and out of control (2.3.3). She did not know how many other ways to find calmness (2.3.4) or to help herself – except through self-harming: “I had to go out of my class all the time … I had to cut myself every day to manage.” The stressful thoughts and feelings during self-harming are understood as alien and difficult to understand, tolerate, or integrate as part of self – something is wrong with me (2.3).

#### Role of Others: Self-Harm Express Being Vulnerable – Important to Fulfill Other's Expectations

Lisa described high expectations for herself of being best, proper, and clever (2.4.1): “I have to be best in my class. I'm a top athlete – doing sport at the highest level. It's so much pressure and expectations and... Further, I must be ready to go to a party and be together with my friends... It's so much! And I live in three different places. I can't manage this anymore.” It is important for Lisa that problems and self-harm must be kept hidden (2.4.2) from her family and friends: “I cut myself on the hips because it's easier to hide with my underwear.” In general, no one should know (2.4.2) about her problems: “I have always been outgoing, happy, and not afraid of anything. Inside I feel different and sad (…) I am good to hide my feelings.” Lisa described how she accepted self-harm as a part of her: “I don't care. It's just how I am.” However, during the interview, when given an opportunity, she could detect feelings related to self-harm such as irritation (at herself) (2.4.3): “I can have a really good time, and then, suddenly, I'm irritated. It's annoying.” Even if she wanted to hide her problems, she appreciated therapy and to speak more openly (2.4.4): “Here at the clinic, I don't have to lie. It's a relief.” Further, Lisa described how self-harm became a sign of being vulnerable (2.4.5): “I started to harm myself because of the bullying and several deaths in my family – I never sat down or cried. I just went on. I never said anything to anyone.” To end self-harm made her aware of requiring help (2.4.5): “I am a person who accomplishes my goals, but to stop self-harm was a difficult task!” The essence of this theme summarizes how important it is to fulfill expectations and how self-harm expresses being vulnerable (2.4).

### Superordinate Theme #3: The Harmed Self – I Am Harmed, and No One Cares

The third superordinate theme and associated themes represent an experience of self-harm dominated by chaos and of being at risk and left alone. Self-harm becomes a way to bring distraction from this chaotic experience and represents something to rely on. Further, self-harm is related to a feeling of being offended, abused, or neglected, and is understood as a legitimate way to show others they are hurt. The self-harming act may concretely express the experienced failure of care – “the harmed self” – and, still, represent a way to prevent a psychic breakdown by re-establishing boundaries. They have given up on being heard, but the behavior of self-harm and involvement in risk-situations are still expressing their distress. The earlier trauma may be re-experienced here and now, again and again, through self-harming.

#### Experiences During Self-Harm: Self-Harm Situations Are Chaotic and Difficult to Remember – It Just Happens

Sophie (17 years old) was referred to the clinic because of depression and suicidal thoughts. She started to harm herself by cutting at 12 years of age. It was hard for her to remember details about situations leading to self-harm (3.1.1): “It's very hard to describe (…) I have a bad memory. Nothing had happened in a way, and no stress, really, and then it just happened.” She described chaotic feelings and thoughts about herself and others before, during, or after self-harm (3.1.2): “To get away from thoughts, for example of being a bad person … the wish to kill yourself or that everything is just chaos.” However, when invited to explore a situation, Sophie remembered being sad earlier that day and a strong feeling of being left alone (3.1.3): “Everything is hopeless and without meaning. It's the same anyway – no one cares. It's frustrating.” Still, she could not relate self-harm to any specific in life (3.1.4): “I don't know how it became like this.” The essence of this theme is summarized as chaotic situations that are difficult to remember – it just happens (3.1).

#### Purpose of Action: Self-Harm Is a Distraction That Makes Difficult Feelings Go Away and Release Something Good – Something to Rely On

Sophie underlined how self-harm was a distraction by attending to the blood and the pain (3.2.1): “You get finished, in a way – I become distracted, and I don't think about it anymore (…) I make myself a “bobble” and feel no pain.” The physical pain and the blood make bad feelings, anxiety, and thoughts go away (3.2.2): “When you cut yourself you don't think of anything else (…). You are only focused on cutting and the blood.” In this way, self-harm releases something good (3.2.3) and could make you addicted (3.2.4): “It's like a drug, in a way – people take it to escape. It gives you a “drug-feeling,” a little happiness. It's an addiction.” Self-harm brings back a feeling of safety through the body (3.2.5): “I did it when I couldn't do anything else. It was something to rely on.” The action of self-harm is highlighted as a distraction that makes difficult feelings go away and releases something good – something to rely on (3.2).

#### Self-Description: Being Offended or Abused and Involved in Risk Situations – No One Cares

Sophie described traumatic situations of violence, assault, and abuse during her childhood (3.3.1): “It was turbulent (…) My mother had physical and mental problems (.) The Child Protective Service was involved since I was 2 years old. They never offered me anything helpful (…) I don't know my father. He was violent and doesn't love me.” Sophie described how she was involved in several risk situations as a child (3.3.2): “I went to school, and then I came back home at 1 a.m. I stole – not because I needed anything, but because it was fun. I went to bars and smoked weed. I had sex with strangers. I was a real slut … I did EVERYTHING at that time. Drugs, you name it.” Sophie was often involved in risk-situations today and didn't know why (3.3.3): “I was reported by the police, laid down in handcuffs (.) I was drunk... I wanted to dance and did a lot of stupid stuff (.) You could have given me LSD – offered by a stranger on the street – and I would have taken it.” She doesn't know why: “I don't know – I'm looking for adrenalin. Taking risks and making bad choices.” In difficult periods, she expresses carelessness and lacked self-support (3.3.4): “I don't give a shit about anything (…) Life is a piece of shit.” She had a hard time believing someone cared for her: “I know my mother cares, but it feels like no one cares.” She described suicidal thoughts always in the background (3.3.5): “It's the first and last thing I think about. When I can't kill myself, I harm myself instead. I can't deal with my life.” The essence of experiencing self during self-harm is summarized as being offended or abused and involved in risk situations – no one cares (3.3).

#### Role of Others: Self-Harm Transform Inner Psychic Pain to Physical Legitimate Pain – A Way to Show I'm Hurt

Sophie described how self-harm changed psychic pain (3.4.1) on the body, which was more tolerable: “I have cut myself SO much, in a way, and it changes from screaming to whispering, in a way, you don't think, because you can't think of two pains at the same time.” During the interview, Sophie reflected on how self-harming could have been a way to tell someone about how troubled she was as a little girl (3.4.2): “My whole body was covered (with scars) … it was insane. Strangely, even people in my own family didn't notice.” Sophie described how adults saw her struggle or self-harming but did not react (3.4.3): “Some saw it … people talked about it, like mom for example – she was very: Are you stupid? You can't do this! She didn't understand … She didn't ask why or – she was just: Don't do it!” Her own reactions to self-harming evoked reflections (3.4.4.) and a possibility to feel sorry for herself: “First, I was indifferent, but then my tears came, and I was so disappointed: what have I done!.” However, she couldn't express herself openly: “To share … it's difficult because I feel vulnerable, and … kind of naked … feel stupid and embarrassed.” She was worried about her impulsivity: “I can “click” and do something without thinking.” Her thoughts about ending her life were quieted by her love for her family: “I have to think about my family. I care about them.” The essence of this theme summarizes how self-harm is experienced as transforming psychic pain to a physical legitimate pain which is interpreted as easier to share – a way to show I'm hurt (3.4).

## Discussion

Strong and fast-changing emotions characterize adolescence. Finding a way to handle, and process difficult feelings and experiences of self is an important developmental task. The present qualitative study underlines how self-harm is experienced as a way to handle inner emotional pain and trauma. The results support the theory of self-harm as a function of affect regulation (Klonsky, 2007; Miller et al., 2019), and are overlapping with results from earlier qualitative studies that highlight self-harm as an attempt to cope with emotional *and* interpersonal problems (McAndrew and Warne, 2014; Sinclair and Green, 2015; Wadman et al., 2018). However, this study highlights qualitative nuances and illustrate patterns in how adolescents experience and represent difficult experiences of self and others during self-harm that are not analyzed systematically across a clinical sample in earlier studies. In the following, self-harm is discussed as closely related to challenges in finding a way to handle, represent, and integrate difficult feelings and experiences, and, regarding developmental challenges of self-identity formation. The qualitative different experiences during self-harm are discussed as emerging self-representations which are integrated parts of self in different degrees.

### Self-Harm – To Handle Difficult Feelings and to Represent Experiences Through the Body

Although all participants shared self-harming, the three superordinate themes illustrate how self-harm may be motivated by qualitative different overwhelming experiences. The results are covering some of the specificities of the subjective experience of self-harm and suicide attempts, especially concerning relationships with the self and with others, which are reported by Grandclerk et al. (2019). Still, the results nuance and exemplify patterns of qualitative essential features in experiences *during* self-harm which may vary among self-harming young people – conflictual *differentiated* feelings and thoughts which evoked guilt (#1), an uncontrollable state of *diffuse* feelings and stressful thoughts which evoked helplessness (#2), or a *chaotic* feeling and state of being abandoned (#3).

Following the theoretical perspective of mentalization (Rossouw and Fonagy, 2012; Luyten et al., 2020), self-harm can express a struggle to regulate *and* to represent intentions, needs, and affect underlying one's own and others' actions. The concrete motoric action of harm toward the body may be understood as *a step* toward a mental representation of difficult feelings and inner pain. These feelings and experiences may be experienced as “alien,” only partly personally integrated or not yet recognized or symbolized in images or words, and, therefore, may be difficult to tolerate (Rossouw and Fonagy, 2012). The physical pain and the physical cut may be a way to represent difficult feelings and experiences that are *integrated to different degrees* – differentiated and conflicted (#1), unknown or diffuse (#2), or an unprocessed chaotic state related to traumatic experiences (#3). In this way, self-harm may be a concrete attempt *to regulate* but also a possibility *to get to know yourself* through the body.

### Self-Harm – An Attempt to Represent Self

The results of this study highlights how experiences during self-harm seem closely related to experiences of the self in different ways. For some, self-harm can be related to self-criticism and self-punishment – *I deserve it* (#1). Self-punishment as the function of self-harm is emphasized in early psychoanalytic studies (turning aggression toward self; Freud, 1917; Parfitt, 2005), and some studies have underlined self-criticism as highly present among self-harmers (Glassman et al., 2007; Hamza et al., 2014; Hooley and St Germain, 2014). The action of harming demands energy and determination and the cut can be a channel for expressing power, destructivity, and anger. Importantly, the interpretation of self-harm as a possible expression of anger and frustration that is conflictual and impossible to express freely and instead is directed toward self is grounded and validated in the participants' descriptions. The concept of *the punished* self (#1) is suggested as one possible emerging self-representation during self-harming.

Self-harm could also be a way to get away from all feelings – *I don*'*t want to feel anything* (#2). Relief from and control over difficult feelings as the function of self-harm is emphasized in the affect regulation theory (Klonsky, 2007; Miller et al., 2019). However, the specific mechanism, which explains how self-harm regulates affect, is not identified (Bentley et al., 2014). More specifically, self-harm is suggested as having an affect regulation function because the person has an autonomous disorder or neuro-cognitive problem (Bentley et al., 2014), or even an impulse control disorder (Favazza, 1998). However, different mechanisms may interact in different degrees in different developmental periods, and we do not know how a person ends up self-harming when there are “a myriad of other behaviors, both functional and dysfunctional, that can serve to fulfill any single intrapsychic or interpersonal need” (Soyemoto, 1998, p. 537). Although self-harm seems to have a function of affect regulation, a study of the capacity of affect integration or mentalization concerning self-harm highlights aspects and differences in a person's way of representing and organizing mental content that may add important knowledge on the *diversity* among self-harmers. Concretely, self-harm breaks or invades the physical skin – the boundary or protective shield of the inner body from an outer world. The blood coming out of an open wound may represent the difficult and alien, and for some may be experienced as the “bad” parts of self. Self-harm can be understood as a way to get control of difficult feelings but may also evoke curiosity for personal needs – *the unknown self* (#2).

Self-harm may even be related to impulsive risk behavior which prevents a psychic breakdown related to an earlier unprocessed trauma of being invaded, forgotten, or assaulted – *I*'*m harmed, and no one cares* (#3). Several studies emphasize how earlier trauma, sexual or physical abuse and neglect in early years are risk factors for self-harm in adolescence (Hawton et al., 2012; Nock, 2014; Miller et al., 2019). The motoric action of self-harm may be a possible link between the private and public domain – an attempt to express and communicate unconscious or non-verbal private content through bodily actions to oneself or others (Lemma, 2010; Yakeley and Burbridge-James, 2018). The scars can even be a sign and identification of inner problems, showing a narrative of their history, a dialogue without words between themselves and their wounds (Gardner, 2001). Lacking someone to support, reflect, or comfort, the body can be used to survive psychologically and to represent experiences of being neglected – *the harmed self* (#3).

The three superordinate themes illustrate conflicting needs, unrepresented affect, or reminders of the trauma, which can be felt as the truth and must be *hidden, controlled, or cut away from self*. The results underline the *private and individual content* that the cut, scars, blood, and wounds invite the person to reflect upon – the need to punish themselves for being angry or bad (#1), the difficulties of expressing and tolerating feelings or vulnerability (#2), or a need for support and care and to process rejection (#3). To develop a stable sense of self and identity requires that a person have the opportunity to experience and explore their own *and* others' feelings and needs (Erikson, 1980; Schore, 2003; Rossouw and Fonagy, 2012). Importantly, the participants in this study described loneliness and they could not bother their parents or friends with difficulties. The destructive action of self-harm could be an attempt to be self-sufficient and independent, *and*, still, a struggle to represent and share inner psychological pain and a call for help from someone who can respond adequately (Motz, 2010; Brady, 2014), a way of social signaling (Nock, 2014), or influencing others to get sufficient support (Klonsky, 2007).

### Clinical Implications

Even though some treatment models show an effect on reducing self-harm, no treatment models help all patients struggling with self-harm, and the rates of dropout are high (Hawton et al., 2015, 2016). It is unclear how specific and common treatment factors, and common elements across treatment models, affect the treatment outcome (Bateman et al., 2015). The three superordinate themes, illustrating personal experiences and purposes of the action during self-harm, underline the importance of exploring the experience and function of self-harm in the patient's everyday life, relational context, and inner world. In treatment, it is important to focus both on symptom reduction and to understand self-harm as part of self-identity formation. Treatment models may in different degrees focus on finding ways to represent and express difficult experiences, on developing alternative coping strategies, or on existential and relational exploration in the therapeutic relationship. The findings of three superordinate themes in the experience of self-harm may increase clinicians' possibility to understand and explore the developmental challenges of self-identity formation with these vulnerable adolescents in addition to affect regulation problems and processing trauma. If the patient feels understood, self-tolerance, and motivation to end self-harm and to explore alternative coping strategies may enhance. Further, knowledge of differences among the three experiences and representations of self could involve some adjustments in treatment-interventions, such as increasing self-reflection and self-compassion (#1), differentiation and integration of affects (#2), or structural care and practical self-support (#3).

### Implications for Society

During adolescence, a person's familial, social, cultural, and digital context can offer different channels to explore and express identity. The girls in this study seem to use their bodies to find reflexivity and some concrete “room” to explore their inner states – what is inside of me? The concrete body can be the primary place for self-identity formation during adolescence (Le Breton, 2017). The increase in self-harm among adolescents, and especially among girls, raises the question of how to represent, express, and share difficult experiences and vulnerability for a young person. The adolescent boy or girl needs relationships and cultural channels to explore inner self, social roles, and social identity – including both the preferred and the difficult parts of self. Internet and social media technology have become important parts of children and adolescents' life and are important ways to seek social acceptance and to gather information but may expose young people to harmful online content (Best et al., 2014). Being exposed or involved in self-harm online content may trigger and normalize self-harming behavior but, interestingly, participants highlight websites with self-harm content as a medium to communicate, experience support from peers, a source for stress alleviation, and ways to express difficult emotions and explore self (Marchant et al., 2017). During adolescence, these websites may for some adolescents be digital communities for sharing and validating vulnerability and to explore “secret” parts of self. The high frequency of self-harming and interaction of self-harm online content may be understood as a signal of difficulties young people may experience in finding ways to express, share or explore important parts of self during adolescence with family, friends, or peers. This knowledge highlights the importance of meeting people who self-harm with a non-judgmental attitude.

### Limitations

The participants consisted of adolescent girls in a clinical context, which could limit the application of the results to girls in a community sample or boys and adults in general. In the present study, there were fewer boys to ask to participate in the clinic. Maybe this reflects the frequency known from survey studies, and maybe there had been more boys to involve if this study included indirect self-harming. Although the findings from the study may be non-transferable, context-dependent, and based on a small sample of participants, the developed concepts developed from the study could be relevant to understand aspects of the phenomenon of self-harm in another context or another sample (Levitt et al., 2017). The concepts can be transferred and discussed regarding knowledge from other studies because of the assumed relationship between data, results, and a theory of the phenomenon. Although the sample included a range of socio-economic status, the sample consisted of mostly a homogeneous ethnic status. Further, treatment experience could have affected their way of making sense of self-harm. However, the analysis is based on the interviews from the beginning of treatment. Focusing on specific situations was useful to obtain their cooperation and to evoke associated personal experiences.

In this study, the interviewer and the principal investigator were the same person. The researcher's bias of perception may influence the interviews and data analysis. To mitigate these challenges, several strategies to manage and to be conscious of the researcher's perspectives were included. During and after the interviews, the main researcher made self-reflective journaling, and initiated dialogue, and gave feedback to the participants to give them a possibility to confirm or correct information. Further, the data-analysis were made together with two senior researchers (see data-analysis), and feedback was sought from a national and international research network on self-harm. Besides, a resource group consisting of young adults with experience of self-harm and treatment (who were not informants in the study) was consulted to see whether the results and the generated concepts were meaningful in addressing the analytic goals (communicative validation; Flick, 2002). They expressed that the results and developed concepts were meaningful and understandable, and emphasized the utility for patients and their families, clinicians, and the general public.

### Future Studies

Further research is needed to see if the results are relevant for adolescent girls in a non-clinical sample and boys and adults in general. It would be of great interest to explore thoroughly the possible similarities and differences between girls' and boys' experiences and trajectories of self-harm. Most studies on sub-groups among self-harmers highlight diagnostic features. In the present study, the three superordinate themes of subjective experience are discussed as ways to represent inner pain and vulnerability as part of the self. It could be interesting to study patterns of experience and self-representation concerning differences in treatment experiences of what is helpful to end self-harm, as well as possible neurobiological differences. One hypothesis could be, following the results of the present study, that knowledge of differences among the three experiences in integration and representations of self-experiences could benefit from some adjustments in treatment-interventions, such as increasing self-compassion (#1), differentiation and integration of affects (#2), or structural care and practical self-support (#3).

## Conclusion

This study highlights three superordinate themes with associated themes concerning essential nuances and qualitative features of experiencing self-harm: 1. The punished self – I deserve it, 2. The unknown self – I don't want to feel anything, and 3. The harmed self – I'm harmed, and no one cares. Self-harm is discussed as both a way to regulate and control difficult feelings and, besides, a way to represent and to get to know yourself. Young girls' experiences during self-harm are discussed as emerging representations of self which are integrated into different degrees – conflictual differentiated feelings and thoughts, uncontrollable diffuse feelings, or a chaotic state of earlier trauma. Self-harm is discussed as part of mental illness *and* according to developmental challenges of self-identity formation during adolescence. This knowledge highlights the importance of how the patients' families, schools, and hospitals understand and meet people who self-harm with a non-judgmental attitude. Further, in treatment, to understand self-harm as part of self-identity formation is of great importance for maintaining improvement in addition to symptom reduction. The sub-types may even inform some important individual adjustments in treatment-interventions such as increasing self-compassion, differentiation and integration of affects, or structural care and practical self-support. Even if treatment models in different degrees focus on self-harm as a function of representing difficult experiences, as affect regulation, or as existential and relational exploration, adversity in interventions and focus of treatment should be weighted in every treatment process because patients are different.

## Data Availability Statement

The datasets generated for this study are available on request to the corresponding author.

## Ethics Statement

The studies involving human participants were reviewed and approved by The Norwegian Regional Committees for Medical and Health Research Ethics (2014/832). Written informed consent to participate in this study was provided by the participants' legal guardian/next of kin.

## Author Contributions

LS has planned the project, collected the data, and led the data-analysis process.

## Conflict of Interest

The author declares that the research was conducted in the absence of any commercial or financial relationships that could be construed as a potential conflict of interest.
